# Comparison of the Trend of Excimer laser refractive surgery in Provinces of Iran between 2010 and 2014

**Published:** 2020

**Authors:** Hassan Hashemi, Mohammad Saatchi, Abbasali Yekta, Marzieh Nojomi, Soheila Asgari, Mehdi Khabazkhoob

**Affiliations:** * Noor Research Center for Ophthalmic Epidemiology, Noor Eye Hospital, Tehran, Iran; ** Noor Ophthalmology Research Center, Noor Eye Hospital, Tehran, Iran; *** Refractive Errors Research Center, Mashhad University of Medical Sciences, Mashhad, Iran; **** Preventive Medicine and Public Health Research Center, School of Medicine, Department of Community Medicine, Iran University of Medical Sciences, Tehran, Iran; ***** Department of Medical Surgical Nursing, School of Nursing and Midwifery, Shahid Beheshti University of Medical Sciences, Tehran, Iran

**Keywords:** excimer laser, refractive surgery, Iran, trend

## Abstract

**Purpose.** The aim of the present study was to compare the trend of excimer laser refractive surgery in different provinces of Iran.

**Methods.** This cross-sectional study was performed in 12 provinces of Iran in 2015 using the data of 2010 to 2014. A total of 28 surgical centers were selected. For each center, one week per season was randomly selected, giving a total of 20 weeks for all seasons of the study years. Then, to estimate the surgical rate in the selected provinces, since 4 weeks were selected each year, the number of operations on the 4 weeks was multiplied by 12.5 to generalize the results to 50 weeks (= 1 year). After applying the weight of each center, the number of refractive operations on each year was divided by the population of the province on that year, and reported per million population.

**Results.** On average, Kermanshah (35.8%, P<0.001) and Tehran (3.1%, P<0.001) had the highest and lowest annual increase and Qom (11.8%, P<0.001) had the highest annual decrease in the rate of excimer laser refractive surgery, respectively. The highest rate was seen in Tehran in 2012 (8885 operations per million population) and the lowest rate was seen in Gilan in 2010 (142 operations per million population). Moreover, the concentration index was 0.25 in 2012, indicating a socioeconomic inequality in the rate of excimer laser refractive surgery.

**Conclusion.** The results of the present study showed an increasing trend in the rate of the excimer laser refractive surgery in 9 Iranian provinces for the first time. Moreover, concerning the inequalities and the higher surgical rate in provinces with a better economic status, it is necessary to expand an insurance coverage and equip more public centers with the instruments and devices required for laser refractive surgery.

## Introduction

According to a WHO report in 2014, uncorrected refractive errors are the most common cause of moderate and severe visual impairment, accounting for 43% of the cases of visual impairment (122 million individuals) worldwide [**1**]. Uncorrected refractive errors are a major cause of low vision or even blindness, and are associated with irreparable consequences for the affected people and their families [**2**]. Among three common treatments for refractive errors, including glasses, contact lenses, and surgery, the patients increasingly prefer permanent methods, such as different surgical techniques. Irregular use of glasses for aesthetic reasons and availability of low-risk surgical procedures are the most important reasons for the popularity of refractive surgery [**3**]. The high incidence of refractive errors in the middle-aged people in Iran [**4**] on the one hand and improved access to different types of refractive surgery on the other hand are predictive of an increase in the rate of refractive surgery as compared to other treatment modalities. Studies in Iran suggested that the rate of excimer laser refractive surgery has increased from 2764 operations per million population in 2010 to 3744 operations per million population in 2012 [**5**]. Although knowledge of the total rate of refractive surgery in the country and the increase in the rate in recent years is important for policymaking in the field of refractive surgery, knowledge of the trend of the surgical rate in each province and the identification of the regions with a decreasing trend despite the increase in the overall surgical rate in the country is of paramount significance. On the other hand, an increasing trend may be observed in some provinces, may be due to either an increase in the prevalence of refractive errors in those provinces or improvement in the knowledge and equipment required for refractive surgery, warranting further studies. The present study was conducted to determine the trend of refractive surgery in 12 provinces of Iran located in different geographical regions with heterogeneous socio-economic statuses and to provide accurate information for future studies in these provinces.

## Methods

This cross-sectional study was conducted in 12 provinces of Iran in 2015 using the available data of refractive surgery from 2010 to 2014. Eight provinces (West Azerbaijan, East Azerbaijan, Kermanshah, Qom, Qazvin, Sistan and Baluchestan, Khuzestan, and Gilan) were selected randomly and the cities in these provinces, that had refractive surgery centers, were listed. The provinces of Tehran, Isfahan, Razavi Khorasan, and Fars were not selected randomly because they are the main centers of refractive surgery in their regions. After identifying the cities, a list of refractive surgery centers in each city was prepared. In cities with more than 2 centers, a number of centers were randomly selected proportional to the population of the city. Finally, 28 centers were selected. **Table 1** shows the proportion of each province. 

**Table 1:** The number of selected and active excimer laser refractive surgery centers in each province

Province name	Number of selected centers	Number of active centers	Sampling weight
West Azerbaijan	1	1	1
Isfahan	5	7	1.4
Khuzestan	1	3	3
East Azerbaijan	3	3	1
Tehran	8	24	3
Gilan	1	1	1
Sistan and Baluchistan	2	2	1
Fars	2	7	3.5
Qazvin	1	1	1
Qom	1	2	2
Kermanshah	1	1	1
Khorasan Razavi	2	5	2.5

**Statistical Analysis**

The number of excimer laser refractive operations during one week per season (a total of 20 weeks for all 5 years of the study) was determined in each selected center to calculate the rate of refractive surgery in different provinces. 4 weeks were selected in each year, the number of operations in the 4 weeks was multiplied by 12.5 to generalize the results to 50 weeks (= 1 year) and obtain the total number of operations in each province in one year. Then, the weight of each center was calculated relative to all centers in that province. **Table 1** shows the method of weighing. The weight of each center was calculated by dividing the number of active centers in the province by the number of centers selected in that province. To calculate the number of operations in the provinces in each study year, the number of annual operations in each center was multiplied by the calculated weight for its corresponding province. Finally, the rate of excimer laser refractive surgery was calculated for each study year and province by dividing the weighted number of operations per year by the population of the province and reported per million population. 

The 95% confidence interval for all point estimated was calculated by Poisson's distribution. The Cochran-Armitage Trend test was used to determine the significance of the trend at a level of 5%. The concentration index was used to determine the role of income and inequality in the rate of refractive surgery using the method proposed by Kakwani, van Doorslaer, and Wagstaff (1997) [**6**]. 

In the health economy, the concentration index is a means to show income-dependent inequality in health variables. This index is twice the level between the concentration curve and the line of justice (line 45 degrees) and has a range between +1 to -1. If there is no difference between the rich and poor people in the outcome variable (in this study, the rate of refractive surgery), it will be equal to zero. If the concentration of the outcome variable is higher in non-prosperous of the community (the poor), the index will be down to the negative one, and if it is higher in the prosperous (rich people), the index will be up to the positive one. In this study, the Conindx command was used to calculate the concentration index and the Glcurve command was used to plot the concentration curve [**6**]. 

Since the most recent income data were collected in 2012, we compared the results of the refractive surgery rate of each province in 2012 with the income of that province in that year. The provincial income was calculated based on the reports of the Statistical Center of Iran on the household budget project. The household income was defined as the Rial value of the goods and services that the household received in return for tasks or capital, or from other sources (pension, assets, transfer payments, etc.). Moreover, incidental incomes, such as the sums received from some institutions like insurance companies (indemnity), were considered the sundry income of the household. Transfer incomes were the sums of money the household received from organizations, institutions, public and private institutes, charity foundations, and other households (i.e. financial aids, donations, etc.). 

## Results

**Fig. 1:**
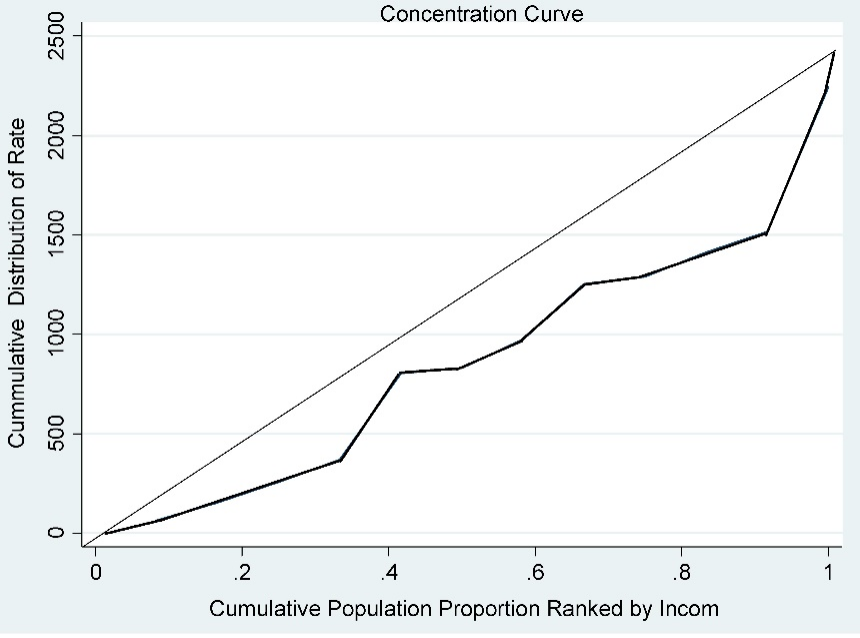
The concentration index graph for the rate of excimer laser refractive surgery in 2012 according to the mean household income in the province

Gilan had the lowest rate of excimer laser refractive surgery in 2010 (350 operations, 1 center), and Tehran had the highest rate in 2011 (34936 operations, 8 centers). **Table 2** presents the total number of operations in each province after applying the weight of each center. Accordingly, 844749 operations were performed in the 12 provinces; Tehran had the highest (507510 operations) and Qazvin had the lowest number of operations (1588 operations). **Table 2** shows the rate of excimer laser refractive surgery in each province in different study years. The highest and lowest rate was seen in Tehran (2012, 8885 operations per million population) and Gilan (2010, 142 operations per million population), respectively. In general, an annual increase of 5.14% was seen in the rate of refractive surgery. Kermanshah (35.8%) and Tehran (3.1%) had the highest and lowest annual increase and Qom (11.8%) and the highest annual decrease in the rate of excimer laser refractive surgery, respectively. An annual increase of 9.8%, 3.6%, 4.8%, 24.1%, 6%, and 21.9% was seen in West Azerbaijan, Khuzestan, East Azerbaijan, Gilan, Sistan and Baluchestan, and Fars, while an annual decrease of 4.3% and 4.7% was seen in Qazvin and Isfahan, respectively. Except for Qazvin (P-value=0.145), the trend of the changes was significant in other provinces (P-value<0.001). The concentration index was used to determine the role of the household income and to evaluate inequality in the rate of refractive surgery. **Fig. 1** shows the concentration index graph between the household income in each province and the rate of excimer laser refractive surgery in 2012. The concentration index was 0.25 with a standard error of 0.16. 

**Table 2:** The weighted number and rate of excimer laser refractive surgery (per million people) in the 12 provinces according to province and year

	2014 number(rate) (95% CI)	2013 number (rate) (95% CI)	2012 number (rate) (95% CI)	2011 number (rate) (95% CI)	2010 number(rate) (95% CI)
West Azerbaijan	1863 (613) (585-641)	2713 (881) (847-914)	2838 (909) (876-943)	3475 (1100) (1077-1151)	3000 (937) (904-971)
Isfahan	23013 (4782) (4720-4844)	29453 (13547) (13392-13702)	33093 (6723) (6651-6796)	25918 (5220) (5156-5284)	30240 (6040) (5971-6108)
Khuzestan	4463 (997) (967-1026)	4463 (985) (956-1014)	6225 (1355) (1321-1388)	5775 (1240) (1207-1271)	5025 (1064) (1034-1093)
East Azerbaijan	3625 (980) (1321-1396)	3688 (990) (958-1022)	4700 (1253) (1217-1289)	4713 (1247) (1211-1282)	4213 (1107) (1073-1140)
Tehran	81756 (6807) (6760-6854)	104807 (8602) (8550-8654)	109358 (8885) (8832-8937)	108720 (8744) (8692-8796)	102869 (8191) (8140-8241)
Gilan	350 (142) (127-157)	575 (232) (213-251)	1025 (411) (385-436)	825 (329) (306-351)	888 (351)
Sistan and Baluchestan	3775 (1505) (1457-1554)	2450 (967) (928-1005)	4125 (1589) (1540-1638)	2000 (752) (719-785)	3400 (1248) (1206-1290)
Fars	6913 (1524) (1488-1560)	14613 (3179) (3127-3231)	13388 (2884) (2835-2933)	15750 (3360) (3307-3412)	19950 (4213) (4196-4315)
Qazvin	0	0	550 (453) (416-492)	525 (428) (392-466)	513 (414) (379-451)
Qom	2800 (2478) (2386-2571)	2250 (1954) (1873-2036)	2125 (1813) (1736-1891)	1750 (1467) (1399-1537)	1850 (1524) (1455-1594)
Kermanshah	425 (220) (199-242)	650 (334) (309- 360)	688 (352) (326-379)	3338 (1701) (1644-1760)	888 (451) (421-481)
Razavi Khorasan	2875 (487) (1406-1605)	4281 (714) (6929-7359)	4813 (791) (7692-8140)	8250 (1337) (1308-1366)	6156 (983) (958-1007)
*No: Number CI: Confidence Interval*					

## Discussion

Our study was the first to evaluate and compare the trend of excimer laser refractive surgery in different provinces of Iran during 5 years. Due to the lack of similar studies, we had some limitations regarding the comparison of the results.

Our findings showed that despite the lowest annual increase in the rate of refractive surgery during 5 years (3% per year), Tehran had the highest rate of refractive surgery per million population. According to previous studies, uncorrected refractive errors account for 22-33.4% of visual impairment in the population of the city of Tehran, as the most important cause [**7**,**8**]. Therefore, the surgical treatment of this visual disorder and the lack of interest in the use of glasses may have contributed to the high rate of refractive surgery in Tehran. On the other hand, Tehran has the highest number of public and private refractive surgery centers (24 centers), which are mostly equipped with the latest technologies and experienced physicians. Moreover, according to the Ministry of Health and Medical Education, many patients travel to Tehran, especially from less developed provinces, to receive medical services, either to bypass the referral system, or due to lack of facilities in their cities [**9**]. Therefore, the high rate of refractive surgery in Tehran is not unexpected considering the high prevalence of refractive errors, available facilities, and massive patient migration. However, the increasing trend of the rate in provinces like Kermanshah, Sistan and Baluchestan, Gilan, and Khuzestan in recent years indicates the good performance of the decentralization process in refractive surgery. 

According to our findings, Kermanshah had the highest growth in the rate of excimer laser refractive surgery (an average of 35%) as compared to other 11 provinces. It should be noted that this province only has one active center. Another interesting finding regarding the highest increase in the rate of refractive surgery in Kermanshah as compared to other provinces was 1701 operations per million population in 2013, which was markedly different from 220-451 operations in other years. Considering the aim of this study and the collected data, further investigations are warranted to understand the reason for this difference in 2013; however, factors like patient migration from neighboring provinces in that period of time may be involved in this difference.

According to our findings, despite the increase in the rate of excimer laser refractive surgery, Qom and Qazvin experienced a decreasing trend in the rate (11.8% and 4.3% decrease, respectively), although a non-significant increase was observed in Qom in 2014 as compared to 2013. It seems that the proximity of Qom and Qazvin to Tehran and patient migration from these two provinces to Tehran may be an important reason for the decreased rate. However, attention should be paid to other factors, including the prevalence of refractive errors and their trends, migration of specialists from and to these provinces, patients’ lack of confidence and trust in fresh specialists, and physicians’ lack of interest to operate in Qom and Qazvin, which require further studies with more specific objectives. Moreover, we noted a 4.7% annual decrease in the rate of refractive surgery in Isfahan despite the activity of 7 centers in this province. It seems that the decrease is secondary to the high rate in 2011 because 4782 to 6723 operations per million population were performed in other study years. 

To confirm the role of income and economics in refractive surgery, our results showed a positive correlation between the household income in each province and the rate of refractive surgery in that province. As discussed in the Results section, the concentration index was below the line of equality (the 45º line), indicating the concentration of refractive surgery in provinces with a better economic status. Concerning the spectrum of the income status of the selected provinces, a marked difference was observed in the rate of refractive surgery between high-income provinces, like Tehran, Isfahan, and Fars, and less developed provinces like Gilan, Kermanshah, and Sistan and Baluchestan. Studies conducted in Iran and other parts of the world have shown a clear relationship between income and visual impairment and confirmed that these disorders, especially refractive errors, are more prevalent in the low-income population [**10**-**12**]. Therefore, the high prevalence of visual disorders on the one hand and the low rate of refractive surgery on the other hand indicate an imbalance in the occurrence of the disease and receiving services, resulting in irreparable consequences in the families living in less developed and low-income regions. Considering the prominent role of correcting refractive errors in promoting the quality of life, in line with the objectives of the Vision 2020, equal distribution of surgical services and specialists should be a priority for health policymakers. 

It is of great importance to note that the current study has not been carried out at an individual level and has been analyzed using aggregate data. Therefore, because of this limitation, the interpretation of income-related inequality between provinces should be made more carefully, because many surgeries in high rate provinces are due to the migration of patients from other provinces to immigrant provinces.

As mentioned earlier, the rate of excimer laser refractive surgery had an increasing trend in almost all the evaluated provinces. However, concerning our knowledge of the high prevalence of refractive errors, it seems that the rates are still lower than expected. Schmack et al. [**13**] reported that although more than 50% of the operations were performed in private centers in Germany, the increase in the rate of refractive surgery in public centers was higher in a 4-year period, from 12% in 2005 to 19.5% in 2008. Since many refractive surgery centers in Iran are private centers and most of them have poor insurance coverage, governmental investment in the public sector, especially university-affiliated centers, is necessary to organize the operations and target groups in different cities. In this regard, providing facilities and equipment for refractive surgery in low-income regions, facilitation of access to refractive surgery by the government and insurance companies, and fair distribution of experienced specialists in different parts of the country through considering incentives to increase their retention in different regions, especially underserved areas, make a major contribution to decreasing visual impairment due to refractive errors. In conclusion, we evaluated, for the first time, the trend of refractive surgery in 12 provinces of Iran with different geographic and economic characteristics. The results showed an increasing trend in 9 provinces during a 5-year period, although the rate is still lower than expected considering the prevalence of refractive errors in the country. With the highest number of the most equipped centers in the country, Tehran had the highest rate of excimer laser refractive surgery but the lowest annual increase in the rate. The rate of refractive surgery was higher in provinces with a better economic status. Therefore, to decrease inequality between less developed and more developed provinces, it is necessary to increase the insurance coverage, equip more public centers with the equipment required for refractive surgery, follow decentralization programs to slow down health migration, and train specialists to offer health services in underserved provinces. 

**Financial Support**

This project was supported by Iran National Science Foundation.

**Conflict of Interest**

The authors declare no conflict of interest.

**Disclosure**

The authors report no conflicts of interest. The authors alone are responsible for the content and writing of the paper. 
